# Draft genome of the cellulolytic bacterium *Acetivibrio clariflavus* strain SA115

**DOI:** 10.1128/mra.01181-24

**Published:** 2025-04-29

**Authors:** Ashley R. Farrelly, Resa B. Nelson, Srilakshmi Garikapati, Jonathan I. Guevara, Javier A. Izquierdo

**Affiliations:** 1 Department of Biology, Hofstra University 174554, Hempstead, New York, USA; University of Southern California, Los Angeles, California, USA

**Keywords:** cellulose, thermophiles, *Clostridium*, lignocellulose

## Abstract

*Acetivibrio clariflavus* strain SA115, a novel isolate obtained from a pulp mill compost pile, is able to utilize cellulose under anaerobic conditions at 55°C. Here, we report the draft genome of this strain.

## ANNOUNCEMENT

*Acetivibrio clariflavus* is a thermophilic, anaerobic bacterium capable of using cellulose as a sole carbon source (1). Strains of *A. clariflavus* can also convert unpretreated lignocellulosic materials to fermentation products while having the distinctive ability to utilize xylose and hemicellulose as carbon sources (2, 3).

*Acetivibrio clariflavus* str. SA115 was isolated from a pulp mill waste compost pile (44.701^o^N, 69.655^o^W) using anaerobic Media for Thermophilic Clostridia or MTC (2) with 5 g/L cellulose (Avicel) as the sole carbon source at 55°C. After a primary enrichment using 5 g/L of compost as inoculum and six consecutive transfers, isolates were obtained on MTC solid media. Strain SA115 was maintained on MTC liquid media with cellulose as the sole carbon source in consecutive transfers. Genomic DNA was extracted from 24 h cultures in cellobiose MTC media using the GenElute Genomic DNA Extraction Kit (Sigma). Sequencing on an Illumina MiSeq DNA sequencer was performed at MR DNA (Shallowater, TX, USA). A DNA library was prepared using a Nextera DNA Sample Preparation Kit (Illumina) following the manufacturer’s user guide. The initial concentration of DNA (5.42 ng/µL) was evaluated using the Qubit dsDNA HS Assay Kit (Life Technologies). A total of 50 ng DNA was used to prepare a library with a final concentration of 12.7 ng/µL. An average library size of 1,020 bp was determined using the Agilent 2100 Bioanalyzer (Agilent Technologies). The library was diluted to 14.0 pM and sequenced paired end for 500 cycles using the MiSeq system (Illumina), generating 2 × 250 bp reads (11.7 M reads). Read trimming and assembly were performed with NGen v12 (DNAStar, Madison, WI, USA), producing 195 contigs (*N*_50_ = 68,191 bp) and a final coverage of 50×. An initial annotation was generated using the NCBI Prokaryotic Genome Annotation Pipeline version 6.5 (4). Default parameters were used for all software unless otherwise specified.

**Fig 1:**
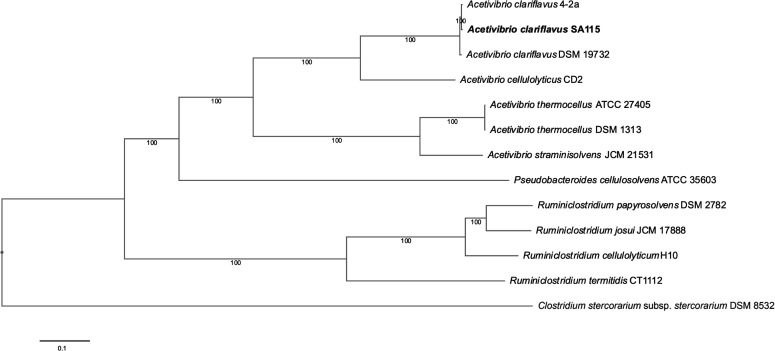
Maximum-likelihood tree based on 100 single-copy genes identified by PGFams constructed with *A. clariflavus* strain SA115 (accession no. JBDHNJ010000000) and related *Acetovibrio* and *Ruminiclostridium* species with accession numbers ASAA00000000, CP003065, AEDB00000000, CP000568, CP002416, BAVR00000000, JQKC00000000, CP119677, BBAA00000000, CP001348, AORV00000000, and CP003992.

The genome of *Acetivibrio clariflavus* SA115 consists of 4,658,389 nucleotides with a GC content of 35.6% and 4,156 predicted genes. 16S rRNA sequencing revealed 99.9% sequence similarity to *Acetivibrio clariflavus* strain 4-2a, accession number FJ808600 (3), using BLASTN version 2.16.0 against the nr/nt database. A maximum-likelihood tree using the codon tree method through the PATRIC platform (5–9) and 100 single-copy genes identified by PGFams shows that *A. clariflavus* SA115 is most similar to other *Acetivibrio clariflavus* strains (Fig. 1). A comparative analysis of strains SA115 and 4-2a reveals that both have an operon for xylose utilization previously reported for strain 4-2a (3). We also identified 27 unique protein-encoding genes in *A. clariflavus* SA115 not found in strain 4-2a, including genes for urea transport and urease activity (accession numbers MEN2775508-MEN2775519) most similar (91.3%–96.0%) to genes in *Acetivibrio thermocellus. A. thermocellus* may use urease activity to withstand lower pH and high alcohol concentration (10, 11), making *A. clariflavus* strain SA115 a promising organism for further study.

## Data Availability

This draft genome has been deposited in GenBank under the accession number JBDHNJ010000000. Raw sequence data are available in the NCBI Sequence Read Archive (SRA) under the accession number SRX26600198, as part of BioProject number PRJNA1108288.
